# Rapid Identification of Cell-Specific, Internalizing RNA Aptamers with Bioinformatics Analyses of a Cell-Based Aptamer Selection

**DOI:** 10.1371/journal.pone.0043836

**Published:** 2012-09-04

**Authors:** William H. Thiel, Thomas Bair, Andrew S. Peek, Xiuying Liu, Justin Dassie, Katie R. Stockdale, Mark A. Behlke, Francis J. Miller, Paloma H. Giangrande

**Affiliations:** 1 Department of Internal Medicine, University of Iowa, Iowa City, Iowa, United States of America; 2 Roche Molecular Systems, San Francisco, California, United States of America; 3 Integrated DNA Technologies, Coralville, Iowa, United States of America; 4 Department of Radiation Oncology, University of Iowa, Iowa City, Iowa, United States of America; University of Helsinki, Finland

## Abstract

**Background:**

The broad applicability of RNA aptamers as cell-specific delivery tools for therapeutic reagents depends on the ability to identify aptamer sequences that selectively access the cytoplasm of distinct cell types. Towards this end, we have developed a novel approach that combines a cell-based selection method (*cell-internalization SELEX*) with high-throughput sequencing (HTS) and bioinformatics analyses to rapidly identify cell-specific, internalization-competent RNA aptamers.

**Methodology/Principal Findings:**

We demonstrate the utility of this approach by enriching for RNA aptamers capable of selective internalization into vascular smooth muscle cells (VSMCs). Several rounds of positive (VSMCs) and negative (endothelial cells; ECs) selection were performed to enrich for aptamer sequences that preferentially internalize into VSMCs. To identify candidate RNA aptamer sequences, HTS data from each round of selection were analyzed using bioinformatics methods: (1) metrics of selection enrichment; and (2) pairwise comparisons of sequence and structural similarity, termed edit and tree distance, respectively. Correlation analyses of experimentally validated aptamers or rounds revealed that the best cell-specific, internalizing aptamers are enriched as a result of the negative selection step performed against ECs.

**Conclusions and Significance:**

We describe a novel approach that combines cell-internalization SELEX with HTS and bioinformatics analysis to identify cell-specific, cell-internalizing RNA aptamers. Our data highlight the importance of performing a pre-clear step against a non-target cell in order to select for cell-specific aptamers. We expect the extended use of this approach to enable the identification of aptamers to a multitude of different cell types, thereby facilitating the broad development of targeted cell therapies.

## Introduction

Nucleic acid aptamers represent an emerging class of pharmaceuticals under development for diagnostic and therapeutic use. Some properties of aptamers that make them promising therapeutic reagents include the intermediate size of aptamers, ease of development, and absence of synthesis constraints associated with small molecule inhibitors and protein-based drugs (e.g., antibodies). Aptamers routinely achieve binding affinities and specificities comparable to therapeutic antibodies. In addition, due to their amenability to modification by medicinal chemistry, aptamers avoid the immunogenicity concerns of protein-based drugs and can be engineered to have optimized pharmacokinetic (PK) and pharmacodynamic (PD) profiles for *in vivo* applications [1], [2]. Furthermore, aptamers can be generated to a range of therapeutic targets more efficiently than is the case for small molecules, high-throughput drug screens or cell-based antibody production.

Isolation of aptamers with affinity and specificity for a target of interest involves iterative rounds of affinity purification and amplification *via* a process termed SELEX (Systematic Evolution of Ligands by EXponential enrichment) [3], [4]. In a typical SELEX experiment, a random sequence oligonucleotide library (with approximately 1×10^15^ sequences) is incubated with a protein target. The protein-bound aptamers are then specifically recovered and amplified by PCR. Single stranded DNA or RNA sequences are then generated from the amplified product and used in a subsequent round of selection. Since the invention of the SELEX process around 1990, many high affinity aptamers that target a wide-range of proteins including transcription factors [5]–[8], cytokines [9], growth factors [10]–[13], proteases [14], [15], serum proteins [16]–[18], cell-surface receptors [19]–[24], cell-adhesion molecules [25]–[28] and viral proteins [3], [29]–[31] have been identified.

Although the traditional SELEX method utilizes a soluble, pure form of the target protein (i.e., recombinant protein), different methods have also been developed that target aptamers to membrane-associated cell surface proteins, termed whole-cell SELEX) [21], [24], [32]–[38]. Like traditional SELEX, whole-cell SELEX is an evolutionary approach, yet whole-cell SELEX allows the selection of aptamers without prior knowledge of specific targets [36]–[39]. Furthermore, whole-cell SELEX can, in principle, generate aptamers to multiple targets in parallel while favoring accessible cell surface epitopes. A major advantage of the whole-cell SELEX method over the traditional *in vitro* SELEX approach is that it facilitates the identification of aptamer sequences that recognize the target (e.g. membrane receptor) in its native milieu; that is, in the context of the cellular membrane. Importantly, this approach overcomes the difficulties in obtaining purified preparations of recombinant membrane proteins [1], [40]. In addition, whole-cell SELEX eliminates the risk that one could select aptamers that will only bind to the purified protein and do not recognize the native form of the protein on living cells.

Aptamers that bind to extracellular targets have high potential for diagnostic and therapeutic applications. For diagnostics, aptamers are used to differentiate among different cell types, such as normal and tumor cells [28]. For therapeutic applications, aptamers that bind to the cell surface can be used directly as activators or inhibitors, or indirectly to direct therapeutics, including small molecule drugs, radioisotopes, toxins or siRNAs, to persist in the vicinity of a specific cell or tissue type [2]. The latter strategy is likely to increase efficacy as well as reduce potential unwanted toxic effects of the therapy. In addition, these reagents can also potentially be used to deliver therapeutics into the cells by increasing the rate of receptor-mediated endocytosis [41].

While whole cell-SELEX has increased the repertoire of aptamers that bind to membrane receptors [21], [33], this methodology does not necessarily select for aptamers capable of accessing intracellular compartments for delivering macromolecules into cells. To isolate RNA aptamers that internalize into target cells, we have recently developed a novel cell-based selection strategy that we refer to as *cell-internalization SELEX* [24]. Our approach has several advantages over the traditional *in vitro* SELEX approach: (1) it favors the isolation of RNAs that bind to receptors in their native state; and (2) it enriches for RNAs that are internalized by the target cell. Using the cell-internalization SELEX approach, we enriched for aptamers that selectively bind to and internalize into HER2^+^-breast cancer cells for delivering therapeutic siRNAs to the cancer cells [24].

A potential downside to the cell-based SELEX approaches as compared to traditional *in vitro* SELEX is that cells are much more complex targets than single recombinant proteins. Two major challenges for cell-based aptamer selections are 1) insufficient methods to monitor the progression of a cell-based selection, and 2) the complexity of aptamer sequences derived from this type of selection. We address these issues by applying a combination of HTS and bioinformatics analysis to the cell-internalization SELEX approach. In this study, we demonstrate the usefulness of this approach by identifying vascular smooth muscle cell (VSMC)-specific internalizing aptamers. We also demonstrate the importance of categorizing aptamers based on structural similarity (structure families), in addition to sequence similarity (sequence families), in order to encourage the identification of functional sequences. In summary, these studies demonstrate the utility of HTS and bioinformatics analysis for facilitating the rapid identification of ‘winner’ sequences from an aptamer selection performed against a complex target. Furthermore, these studies have resulted in several VSMC-specific internalizing RNA sequences that could be used to deliver siRNAs or other small molecule drugs specifically to VSMCs.

## Results

### Enrichment of VSMC-specific internalizing RNA aptamers

**Figure 1:**
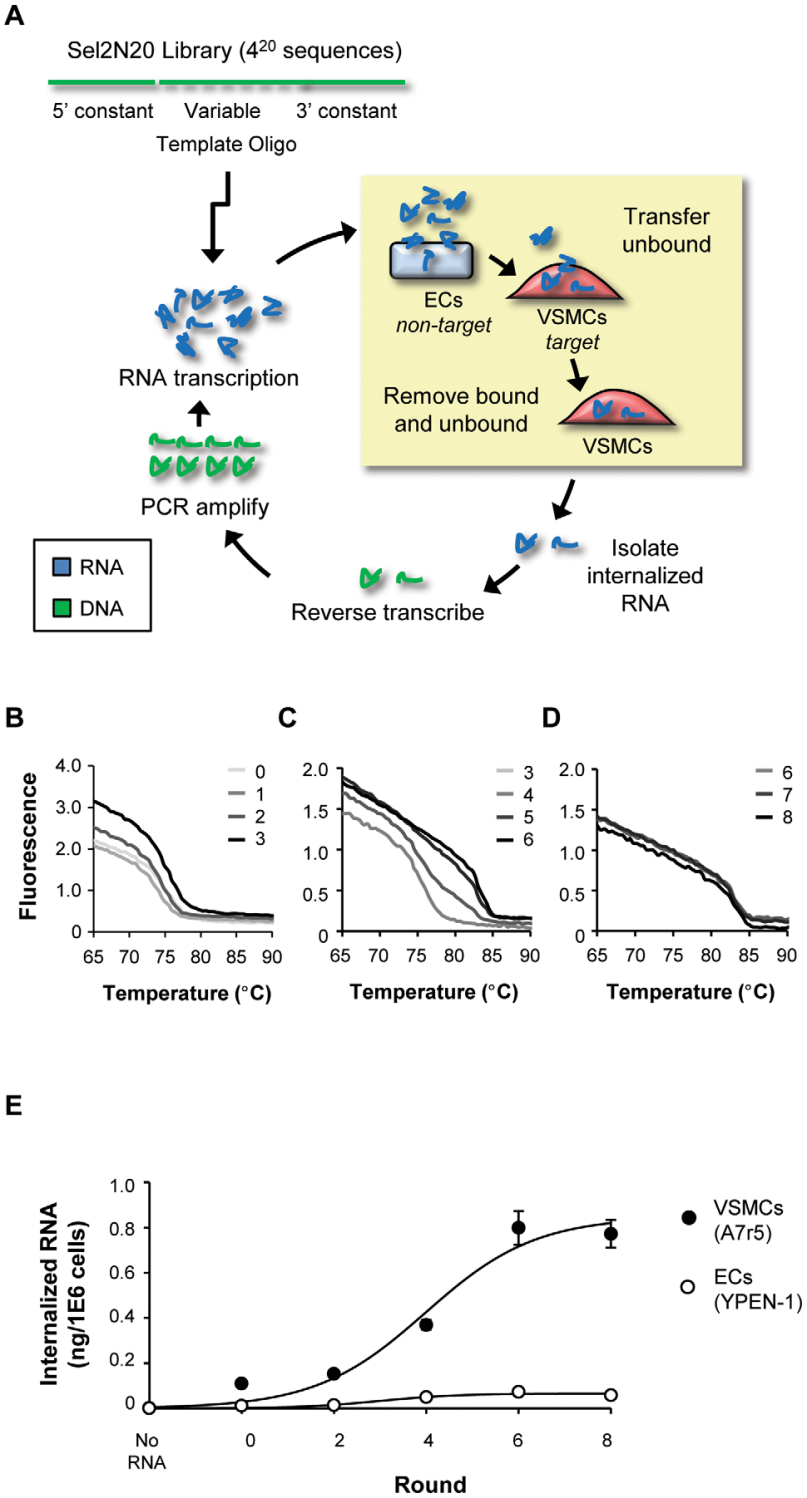
Cell-Internalization SELEX (systematic evolution of ligands by exponential enrichment). (**A**) Schematic of the methodology used to isolate aptamers that specifically internalize into vascular smooth muscle cells (VSMCs; *target*). Eight rounds of selection were performed to enrich for RNA aptamer sequences that selectively internalizes into the target VSMCs. Non-specific aptamers were removed by pre-clearing against endothelial cells (ECs; *non-target*). (**B–D**) Progression of the selection and complexity of the RNA pools was monitored using a DNA melt assay at (**B**) rounds 0–3, (**C**) rounds 3–6 and (**D**) rounds 6–8. (**E**) The RNA pools at each round of selection were tested for internalization into VSMCs and ECs using quantitative RT-PCR (RT-qPCR). These data were normalized to an internal RNA reference control for the PCR and to cell number.

Aptamers that selectively internalize into vascular smooth muscle cells (VSMCs; A7r5) were enriched using the *cell-internalization SELEX protocol* [24] (**Figure 1A**). The cell-internalization SELEX protocol involves incubating pools of RNAs from an RNA library with a *non-target* cell (negative selection) and a *target* cell (positive selection) (**Figure 1A**). Rounds 1 through 3 of selection were performed against target VSMCs (A7r5) using positive selection criteria (**Table 1**). RNA was incubated with cells for 90 minutes, based on a predetermined time for maximizing RNA internalization into these cells (**Figure S1**). To enable the identification of VSMC-specific sequences, a negative selection step against endothelial cells (ECs; YPEN-1) was introduced at rounds 4 through 8 of selection (**Table 1**). Importantly, to enrich for RNA aptamers that internalize into the target VSMCs, we introduced a stringent salt wash to remove any unbound RNA and to reduce surface-bound RNAs.

**Table 1:** Selection conditions.

		Pre-clear		Internalization	
Round	[RNA]	Cell Line	Time	Cell Line	Time (min.)
**1**	150 nM			A7r5	90 min.
**2**	150 nM			A7r5	90 min.
**3**	150 nM			A7r5	90 min.
**4**	150 nM	1× YPEN-1	20 min.	A7r5	60 min.
**5**	150 nM	1× YPEN-1	20 min.	A7r5	60 min.
**6**	150 nM	1× YPEN-1	20 min.	A7r5	60 min.
**7**	150 nM	2× YPEN-1	2×10 min.	A7r5	30 min.
**8**	150 nM	2× YPEN-1	2×10 min.	A7r5	30 min.

We monitored the progression of the selection by measuring the complexity of the RNA pools at each round of selection using a modified DiStRO DNA melt assay [42] (**Figure 1B-D**). The DNA melt assay allows for rapid and cost-effective analysis of selection progression and can be easily applied to complex cell-based selections. In this assay, the DNA from a given selection round (from the PCR amplification SELEX step) is heated at high temperatures (melted) and, as the temperature is decreased, the efficiency of re-annealing of the melted DNA is measured by SYBR green fluorescence. A relative loss in library complexity is indicative of a shift in the DNA melt curve towards higher temperatures. A significant drop in library complexity was observed between rounds 2 and 3 of selection as evidenced by the shift in the round 3 DNA melt curve towards higher temperatures, compared to the DNA melt curves of rounds 0 through 2 (**Figure 1B**). This initial drop in library complexity was an indication that additional selective pressures could be introduced to further enrich for VSMCs-specific RNA aptamers. Thus, a negative-selection step (pre-clear against YPEN-1 cells) was introduced at round 4 of selection (**Table 1**). In addition, to enrich for RNA aptamers that are more rapidly internalized into VSMCs (A7r5), we reduced the time the RNA pool was incubated on cells from 90 minutes to 60 minutes (**Table 1**). These selection pressures were maintained for three consecutive rounds (rounds, 4, 5 and 6) until no further changes in library complexity were observed (**Table 1** **and** **Figure 1C**). At this point, we introduced additional selective pressures into each round: a second negative-selection step and a shorter incubation time for the RNA pools with VSMCs (**Table 1**). Interestingly, a DNA melt assay performed on rounds 6, 7 and 8 of selection indicated no further increase in convergence (**Figure 1D**). These data suggest that selection convergence was achieved at round 6 and that further rounds of selection may not be necessary.

We next verified cell-specific internalization of the RNA pools at each round of selection (**Figure 1E**). RNA from rounds 0, 2, 4, 6 and 8 of selection was incubated with either VSMCs (A7r5) or ECs (YPEN-1). Those RNAs that internalized into the cells were recovered by TRIzol extraction and quantified using RT-qPCR [24]. The absence of RNA was used as a negative control in this assay. Importantly, cell-specific internalization was achieved as early as round 4 of selection (**Figure 1E**). Maximum internalization into VSMCs was attained at round 6 with no significant increases in VSMC internalization in subsequent rounds (**Figure 1E**). These data are in concert with the data generated from the DNA melt assay (**Figure 1B–D**) and suggest that selection convergence for VSMC-specific internalizing RNAs occurred at round 6 of selection.

**Figure 2:**
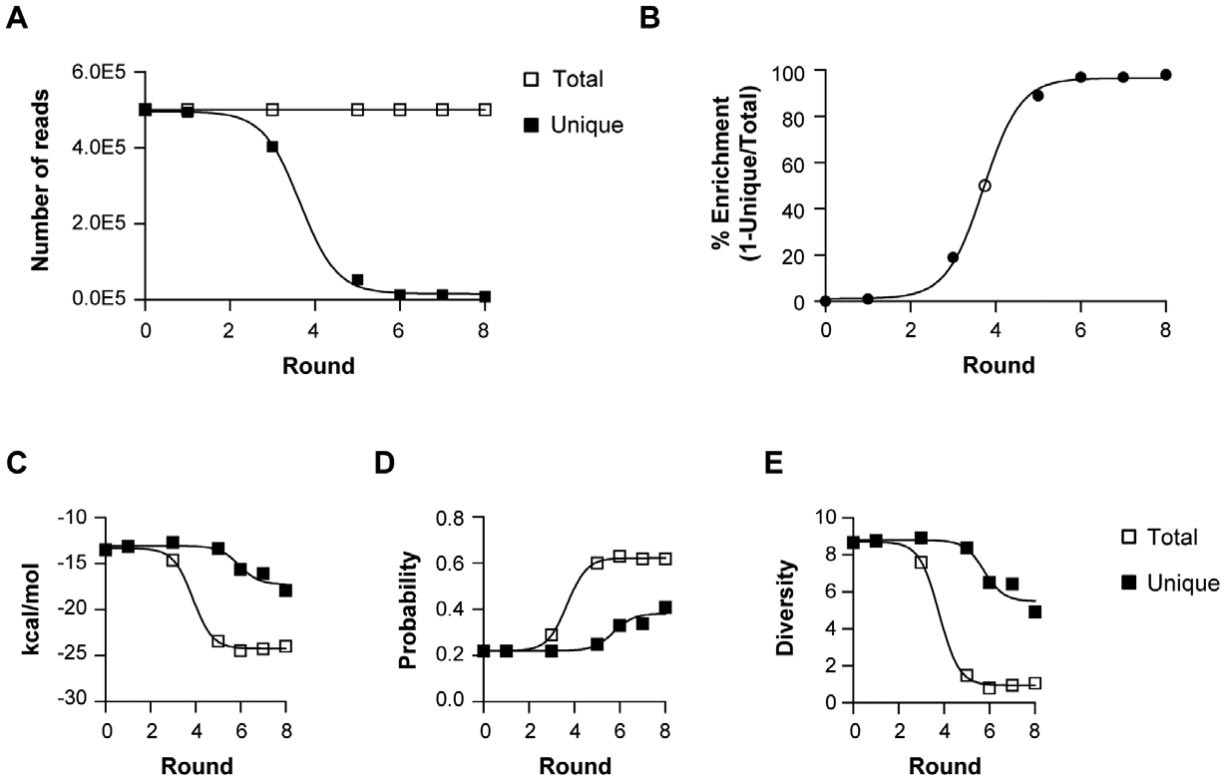
Assessment of selection progression. (**A**) RNA aptamer pools from rounds 0, 1, 3, 5, 6, 7 and 8 were sequenced using Illumina sequencing technology. The number of unique reads (black squares) and total reads (white squares) was determined at each round of selection. (**B**) % Enrichment at each round (black circle) was determined by the formula (% Enrichment = 1-Unique/Total). A sigmoidal curve fit was used to determine the round at which 50% sequence enrichment was achieved (white circle). The average (**C**) minimum free energy (**D**) ensemble probability and (**E**) ensemble diversity at each selection round was calculated for the unique reads (black squares) and for the total reads (white squares) using RNAfold secondary structure prediction algorithm.

HTS, using Illumina sequencing technology, was performed to enable the bioinformatics analysis of millions of sequence reads from selection rounds 0, 1, 3, 5, 6, 7 and 8. We obtained a total of 2,605,039 raw reads from the sequenced rounds (**Table S1**). These raw reads were filtered based on the RNA library constant region sequences. After filtering, 2,546,770 total reads were obtained. Of the total filtered reads, 1,425,964 were unique reads (**Table S1**). After normalizing to total reads (white squares) at each round of selection, the number of unique reads (black squares) was decreased with each subsequent round (**Figure 2A**). This decrease in the number of unique reads observed at later rounds of selection (rounds 4 through 8) is indicative of a decrease in library sequence complexity and an increase in library sequence enrichment.

Next, we estimated the % Sequence Complexity at each round of selection by determining the percent of unique reads relative to the total reads (Unique/Total) (data not shown). Sequence Enrichment (% Enrichment) was then determined by taking the complement of % sequence complexity (1-Unique/Total) (**Figure 2B**). % Enrichment reached a plateau at round 6 of selection with no further change observed in subsequent rounds. As shown in Figure 2B, the most pronounced changes in % Enrichment occurred between selection rounds 3 and 5. Specifically, sequence enrichment approximated 50% by round 4 of selection (**Figure 2B**). Taken with the data in Figure 1, these findings confirm that selection convergence was achieved.

We next examined RNA structural complexity/diversity by performing secondary structure predictions of the unique and total reads at each round of selection. The RNAfold algorithm of the Vienna Package (v 2.0.0) [43], [44] was used to generate the secondary structure predictions. First, we determined the minimal free energy (kcal/mol) (**Figure 2C**) and ensemble free energy (data not shown) of the predicted secondary structures with the highest probability. Interestingly, we observed a decrease in both minimal free energy (kcal/mol) (**Figure 2C**) and ensemble free energy (data not shown) with each progressive round of selection. The decrease in minimal free energy (kcal/mol) for the total reads (white squares) was more pronounced compared to that of the unique reads (black squares). These data suggest that the selection scheme enriched for RNA sequences with higher structural stability as compared to non-selected sequences found within round 0, which have a lower structural stability. The decrease in minimal free energy (kcal/mol) stabilized between selection rounds 4 and 6 (**Figure 2C**). These data are in agreement with the data in Figures 1 and 2B, and suggest that structural convergence may have occurred as early as round 4 of selection.

We hypothesized that the observed decrease in minimum free energy (kcal/mol) in later selection rounds may have resulted from loss of structural diversity. Therefore, structural diversity was assessed by determining the probability (ensemble probability) of the most likely structure for a given sequence (**Figure 2D**) and the diversity (ensemble diversity) of structures that a given sequence may assume (**Figure 2E**). Ensemble probability (**Figure 2D**) and ensemble diversity (**Figure 2E**) was determined for both the unique reads (black squares) and total reads (white squares) at each round of selection. We observed a progressive increase in ensemble probability for both the unique reads (black squares) and total reads (white squares) (**Figure 2D**). However, as expected, the increase in ensemble probability for the unique reads (black squares) was more pronounced compared to that of the total reads (white squares). We observed a concordant decrease in ensemble diversity with progressive rounds of selection for both the unique (black squares) and total (white squares) reads (**Figure 2E**). As expected, the decrease in ensemble diversity was more pronounced for the unique reads (black squares) than for the total reads (white squares). Together, these data suggest that the selection converged towards fewer possible structures with higher structural probability and stability.

### Bioinformatics analysis of HTS data from selection rounds

**Figure 3:**
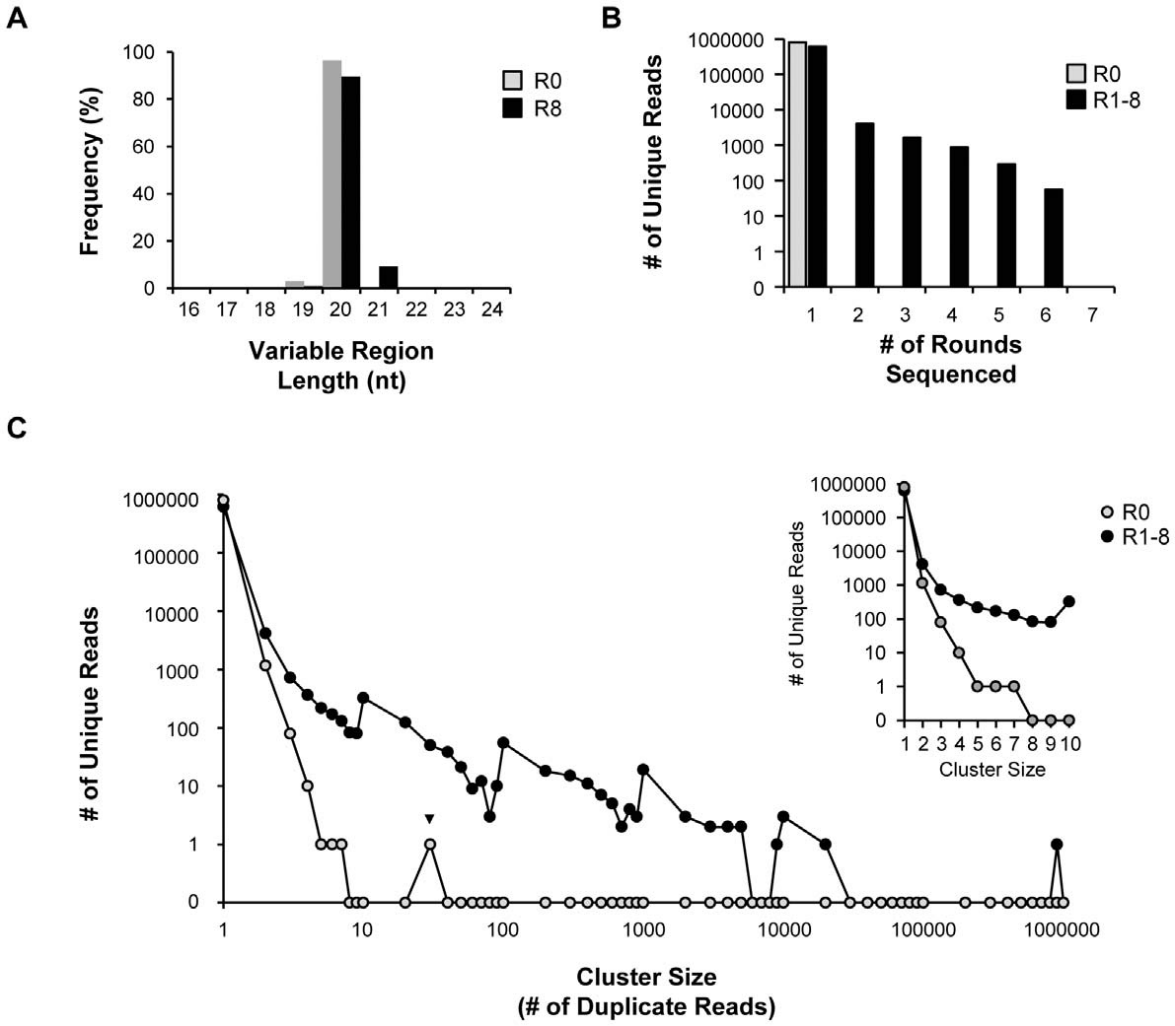
Bioinformatics analysis of high-throughput sequence data from selection rounds. (**A**) The RNA sequences from rounds 0 (gray bars) and 8 (black bars) of selection were examined for frequency of variable region nucleotide (nt) length (ranging from 16 nt–24 nt). (**B**) Number of unique reads from round 0 (gray circles) and rounds 1–8 (black circles) *vs.* number of rounds sequenced. (**C**) Number of unique reads from round 0 (gray circles) and rounds 1–8 (black circles) *vs.* cluster size. A single sequence (▾) containing a string of cytosine was found 30 times within round 0.

Previously, we reported that nucleotide (nt) deletions or insertions within the variable region of a DNA or RNA SELEX library can occur during the course of a selection [24]. Therefore, we examined the variable region nucleotide length of unique reads from selection round 0 (gray bars) and round 8 (black bars) (**Figure 3A**). As expected, the mode variable region length was 20 nucleotides for both round 0 (gray bars) and round 8 (black bars). However, the average variable region length was significantly (*p*<0.001) higher in round 8 (black bars) compared to round 0 (gray bars) (**Figure 3A**). The increase in the average variable region length observed in round 8 (black bars) was most likely due to an increase in frequency of RNA sequences with a 21-nucleotide variable region length. Interestingly, we also observed a higher frequency of sequences with 19-nucleotide variable region length in round 0 which were not present (or likely, were not selected) in later rounds (**Figure 3A**). Based on these observations, we included sequences with variable region lengths ranging from 18 to 22 nucleotides in our subsequent analyses.

We next analyzed the high-throughput sequencing data to separate true-selected sequences from non-selected sequences. We postulated that rounds 1 through 8 would contain a mixture of true-selected sequences and non-selected sequences, while non-selected sequences would be predominantly found in round 0. First, we examined the recurrence of a given unique read between sequenced rounds by comparing the number of unique reads from round 0 and rounds 1–8 to the number of rounds sequenced (i.e., 7 rounds: rounds 0, 1, 3, 5–8) (**Figure 3B**). As expected, all unique reads analyzed were present in at least one sequenced round (**Figure 3B**). By contrast, none of the unique reads from rounds 1 through 8 were found in round 0, suggesting that complete coverage of the round 0 library was not achieved through Illumina sequencing, even after sequencing the round 0 library twice (**Table S1**). Importantly, thousands of unique reads from rounds 1 through 8 where found in two or more rounds, with many unique reads found in as many as six rounds (**Figure 3B**). These data suggest that ‘true-selected’ sequences are more likely to appear in multiple rounds compared to non-selected sequences. Furthermore, the *number of rounds* in which a unique read is found may be used as a cut-off to separate ‘true-selected’ sequences from non-selected sequences. In this case, we favored a low-stringency cut-off. For example, we considered a ‘true-selected’ sequence to be a sequence that was present in at least two or more rounds of selection.

Next, we reasoned that an additional measure of a true selected sequence would be its *cluster size,* which is the number of duplicate sequence reads in each round. Thus, we compared the number of unique reads within round 0 and rounds 1–8 to *cluster size* (**Figure 3C**). The most represented *cluster size* for unique reads within rounds 1–8 and round 0 was a *cluster size* = 1 (**Figure 3C**). As anticipated, unique reads in round 0 are represented by small cluster sizes (<10 duplicate reads), such that only one unique read within round 0 had a *cluster size* greater than eight reads (**Figure 3C**). However, this unique read contained a continuous string of cytosine and thus was likely a sequencing error that was amplified thirty times (30 reads; ▾). In contrast, unique reads in rounds 1 through 8 are represented by large cluster sizes (>10 duplicate reads) (**Figure 3C**). These data suggest that *cluster size* can be used to separate ‘true-selected’ sequences from non-selected sequences. In this case, the initial substantial difference in *cluster size* between unique reads detected in round 0 and rounds 1–8 was observed at *cluster size* = 3 (**Figure 3C** **,** see inset).

Taken together, the *number of rounds* in which a given sequence is found (**Figure 3B**) and its *cluster size* (**Figure 3C**) may be combined to separate ‘true selected’ sequences from non-selected sequences. Based on these analytical parameters, a ‘true selected’ sequence from the cell-based VSMC selection described herein is more likely to be present in two or more rounds and have a *cluster size* of 3 reads. Thus, for subsequent analyses, if a sequence was detected in two or more rounds and had a cluster size of at least three reads, it was considered a ‘true selected’ sequence.

### Edit and tree distance analyses

**Figure 4:**
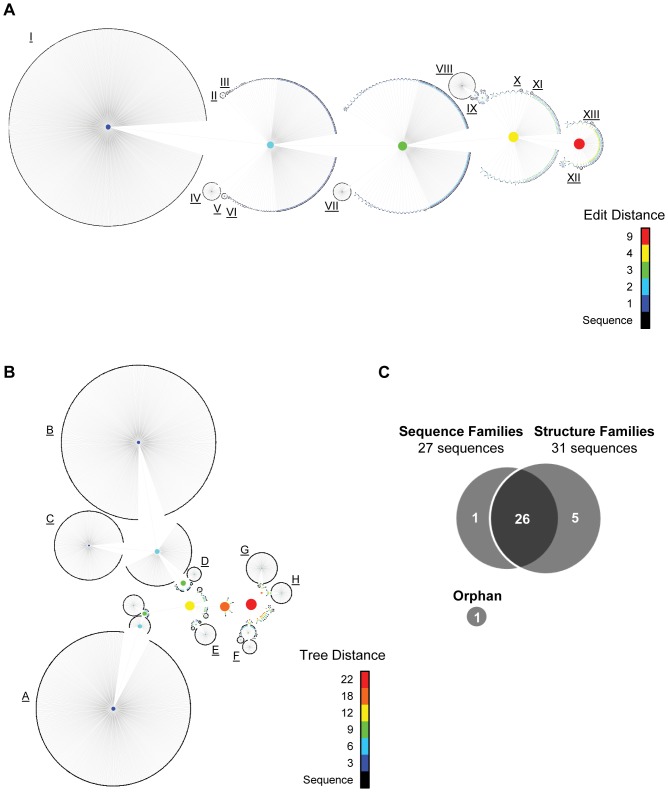
Bioinformatics analysis of RNA aptamers to identify related sequence and structure families. (**A**) RNA aptamer unique sequences (black) are connected to nodes of increasing edit distance (1–9; blue to red color scale). Related sequence families were identified as RNA aptamer sequences that connected by an edit distance of 1 (I–XIII). (**B**) RNA aptamer sequences (black) were connected to nodes of increasing tree distance (0–18; blue to red color scale). Related structure families were identified as RNA aptamer sequences with structures connected by a tree distance of 3 to 6 (A–H). (**C**) Venn Diagram of RNA sequences identified based on the edit distance (27 sequences) and tree distance (31 sequences) analyses. Orphan: a highly represented sequence that did not fall into a sequence or structure family.

Candidate aptamers derived from selection efforts are often categorized based on sequence homology [24], [45] and sequence motifs [8], [46]. We have expanded these analyses to include a novel pairwise comparison of each aptamer sequence using the concept of *edit distance* (**Figure 4A**). Edit distance is defined as the number of changes (substitution/insertion/deletion) necessary for two sequences to become identical. For example, closely-related sequences have a low edit distance, while unrelated or loosely-related sequences are denoted by a high edit distance.

We next determined the edit distance for sequences within rounds 1–8 that were categorized as ‘true selected’ sequences based on the analyses described in Figure 3B and C. A total of 2312 unique reads (**Database S1**), representing 1,123,533 total reads, were analyzed for edit distance (**Figure S3;** output for edit distance = 1 shown) by the program process.seqs (**Figure 4A**). As seen in Figure 4A, all unique sequences interconnect at edit distance = 9 (red node). Unique sequences that interconnect at edit distance = 1 (blue), 2 (cyan), 3 (green), 4 (yellow), and 9 (red) are shown (**Figure 4A**). The most significant clustering of sequences was observed at an edit distance of 1. The dendrogram in Figure 4A was used to identify families of related sequences and to determine how far apart (in edit distance) the sequence families were from each other. At each edit distance node, the robustness of clustering of related sequences was determined using ClustalX multiple sequence alignments (see Methods for details). From these alignments, 13 distinct sequence families (I–XIII) at 1 edit distance apart were established (**Figure 4A**).

Although selected aptamers are typically categorized based on sequence similarity, we asked whether selected aptamers could also be analyzed and categorized by structural similarity. The secondary structure with the highest probability for each of the 2312 unique reads (1,123,533 total reads) was predicted using RNAfold. Each unique read was assumed to have only a single structure, which is supported by the data in Figure 2D and E that suggest that selected sequences have a higher structural probability and low structural diversity, respectively, when compared to non-selected sequences. A pairwise comparison for each structure was performed using the concept of *tree distance,* which describes the relatedness of two structures by calculating the dissimilarity between two structures. Analogous with edit distance, closely-related structures have a low tree distance, while unrelated or loosely-related structures are denoted by a large tree distance. Unique sequences were analyzed for tree distance (**Figure 4B**). The data output derived from tree distance = 3 analysis is shown (**Figure S4**). All of the predicted structures connected with tree distances ranging from 0 (representing an identical structure of a different sequence) to 22 (red node). LocARNA multiple sequence/structure alignments of the predicted RNA structures at the various tree distances were used to establish structure families (see Methods for details). Eight structure families (A–H) were identified and ranged from a tree distance of 3 to a tree distance of 6 (**Figure 4B**).

Next, single representative RNA aptamer sequences were selected from each sequence (**Figure 4A**) or each structure family (**Figure 4B**) based on the following parameters: (1) *fold enrichment* [8], [46], (2) ‘*rising*’, defined by the increasing trend in read number over progressive rounds of selection, (3) *read number,* defined by total reads in a given round and (4) *rate enrichment,* defined by the change in *read number* over change in round number. These parameters were applied to the following rounds: rounds 1–8, 1–3, 3 through 8 and 6 through 8, based on the selection conditions described in Table 1. The analysis of the rounds based on the above parameters resulted in a total of 27 representative aptamer sequences derived from the 13 edit distance sequence families and a total of 31 representative aptamer sequences derived from the eight tree distance structure families (**Figure 4C**). Interestingly, while 26 out of 32 the RNA sequences were identified by both methods, some aptamer sequences were only identified by the edit distance analysis (1 out of 32) or the tree distance analysis (5 out of 32) (**Figure 4C**). In addition, a single highly represented sequence denoted as ‘*orphan*’ (**Figure 4C**) did not group with any of the families derived from the edit distance or tree distance analyses. This sequence comprised a total of 506 reads and was chosen, along with the other 32 sequences, for a total of 33 single RNA aptamers for subsequent analysis.

### Internalization of single aptamers into VSMCs

**Figure 5:**
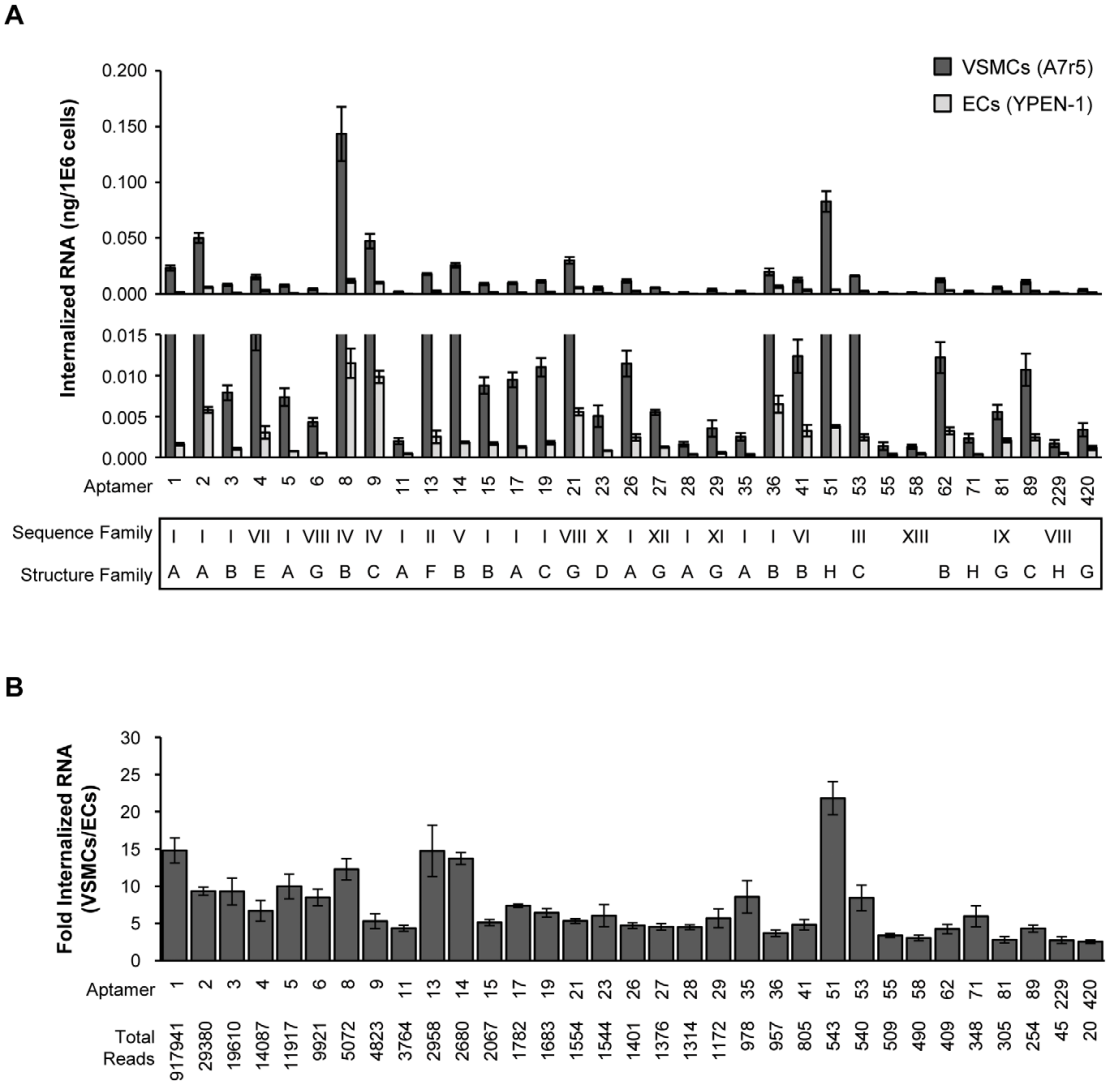
VSMC-specific internalization of candidate RNA aptamers. (**A**) Internalization of individual RNA aptamer sequences derived from the bioinformatics analysis in Figure 4 was assessed in both VSMCs and ECs by RT-PCR (RT-qPCR). The data are normalized to a reference control RNA and to cell number. Internalized RNA data (ng/1,000,000 cells) are plotted on a full scale (top panel) as well as on an expanded scale (middle panel). Table (bottom panel) indicates the sequence and/or structure family of each RNA aptamer sequence. (**B**) Relative fold internalization (VSMCs/ECs) was determined for each RNA aptamer sequence.

To evaluate aptamer internalization, the 33 individual RNA aptamers identified in Figure 4 were incubated with either VSMCs (A7r5) or EC (YPEN-1) cells and the RT-qPCR fold internalization into VSMCs over ECs was calculated after recovery of aptamers (**Figure 5A, B**). All tested aptamers internalized preferentially into VSMCs (A7r5) compared to ECs (YPEN-1) (**Figure 5A**), though it should be noted that the degree of internalization varied from aptamer to aptamer. Approximately 82% of all screened aptamers displayed in a 4-fold greater internalization into VSMCs compared to ECs. Many of the highest internalizing aptamers were identified by both the edit distance and the tree distance analyses (**Figure 5A**). The most represented aptamer, #01 (917,941 total reads) was among the better internalizing sequences (14.8±1.7 fold) (**Figure 5 B**). Interestingly, the aptamer with the highest fold internalization, #51 (21.8±2.2 fold) was identified only by the tree distance analysis but not the edit distance analysis (**Figure 5A**). The ‘*orphan*’ aptamer #55 displayed the poorest internalization (fold internalization <4). Together, these data highlight the importance of performing a tree distance analysis in addition to categorizing aptamers based on sequence similarity. Importantly, these data also suggest that aptamers that do not fit into either edit distance or tree distance families are likely to be ‘junk’ sequences and should not be included in further analyses.

Next, we performed a fluorescent-based ‘plate reader’ assay to confirm specific binding and internalization of our selected aptamers into VSMCs (**Figure S5 and Materials S1**). We assessed binding/internalization of two independent fluorescently-labeled aptamers (#51 and #420). As observed with the RT-qPCR assay, aptamer #51 is a strong cell-specific internalizer, while aptamer #420 is a weak cell-specific internalizer (**Figure S5**). Together, these data confirm that the aptamers selectively internalize into VSMCs vs.ECs.

We used correlation analysis to determine the dependence of fold internalization (into VSMCs) and the following parameters: (1) fold enrichment [8], [46], (2) *rising* (trend of increasing read number across progressive rounds), (3) rate enrichment and (4) read number (**Tables 2** **and** **3**). The correlation analysis determines the dependence between two variables as defined by a correlation coefficient (r) that has a value between −1.0 and 1.0. For example, two variables with a cooperative dependence will have a positive correlation coefficient (r>0), whereas two variables with an opposing dependence will have a negative correlation coefficient (r<0). The correlation analysis also calculates the statistical significance (*p*-value) of the relationship between two variables as described by the correlation coefficient.

**Table 2:** Correlation coefficient (r) for fold internalization vs. *fold enrichment, ‘rising’* and *rate enrichment*.

Rounds	*Fold enrichment*		*‘Rising’*		*Rate enrichment*	
	r	*p*	r	*p*	r	*p*
**1–3**	0.20	0.341			0.02	0.916
**1–8**	**0.52** *	**0.010**	0.27	0.195	**0.54** *	**0.007**
**3–8**	**0.47** *	**0.021**	0.36	0.084	**0.50** *	**0.013**
**6–8**	0.35	0.095	0.18	0.397	0.26	0.215

* *p*<0.05.

**Table 3:** Correlation coefficient (r) for fold internalization vs. *read number*.

Round	r	*p*
**1**	−0.07	0.739
**3**	0.00	0.995
**5**	**0.43** *	**0.037**
**6**	**0.55** *	**0.005**
**7**	**0.69** *	**0.000**
**8**	**0.62** *	**0.001**
**Total**	**0.70** *	**0.0001**

* *p*<0.05.

Significant positive correlation coefficients (r>0 and *p*<0.05) were obtained when comparing fold internalization to fold enrichment for rounds 1 through 8 and rounds 3 through 8 but not for rounds 1 through 3 and rounds 6 through 8. Similar results were obtained when comparing fold internalization to rate enrichment (**Table 2**). These results suggest that cell-specific internalizing aptamers are enriched after the inclusion of a negative selection step (round 4) and that further enrichment occurs after the selection has converged (rounds 6 through 8) (**Table 2** **and** **Figure 1B and C**). In general, fold internalization positively correlated with *fold enrichment*, *read number* and *rate enrichment* only after round 3 of selection, when the negative selection (performed against ECs) was introduced (**Table 1**). In contrast, no significant correlation was found between fold internalization and *rising* (**Table 2**). These data suggest increasing read number between rounds is not sufficient to denote specific internalization. These data collectively suggest that the above parameters coupled to selection conditions should be analyzed in order to facilitate the identification of ‘winner’ sequences.

## Discussion

The identification of candidate aptamer sequences within an enriched library is slowly shifting from traditional cloning and sequencing approaches to the use of HTS [8], [18], [24], [45]–[48]. In spite of this trend towards more sophisticated aptamer identification approaches, the computational tools for the downstream analysis of millions of sequence reads, which result from HTS efforts, are still being streamlined. Here we describe a novel approach that couples HTS with bioinformatics tools to facilitate the identification of over 30 ‘winner’ RNA sequences from a complex, cell-internalization aptamer selection. To identify candidate aptamer sequences, high-throughput sequence data from eight rounds of negative and positive selection were analyzed using these novel methods. Metrics for determining % Enrichment confirmed the experimental cell-internalization data suggesting the selection was complete after eight rounds of selection. Millions of ‘true-selected’ sequences were separated from the non-selected ones using metrics based on the *number of rounds* a sequence was present in and on the *cluster size* for each sequence. The metrics analysis for selection enrichment made it possible to sort through millions of reads and rapidly eliminate those sequences that were not selected or were present in the dataset as a result of sequencing errors. All the unique reads that comprised the ‘true-selected’ sequences were then clustered based on either sequence similarity using edit distance analysis or structural similarity using tree distance analysis. Importantly, these analyses resulted in the identification of sequences that would otherwise have been missed by conventional means, that is, by selecting only the top sequence reads.

The approach described herein, is of importance in light of the rise in complex target SELEX, in which aptamers are directly selected against complex protein mixtures, cells, or even whole organisms [24], [33], [36], [49]–[53]. Although these methods are still in their infancy relative to the original *in vitro* SELEX method, many of the cell-specific aptamers generated so far have served well as neutralizing ligands [21], real-time detection probes [54], [55], as well as internalizing escorts [24]. Particularly impressive, are selections performed against whole organisms (*in vivo*-SELEX) that include the isolation of RNA aptamers that recognize African trypanosomes [51] and *Mycobacterium tuberculosis* [52]. These aptamers have the potential to target biomarkers on the surface of the parasite or bacterium and as such might be modified to function as novel drugs against these unicellular organisms. While African trypanosomes and *Mycobacterium tuberculosis* are examples of simple, unicellular organisms, complex *in vivo-*selections have also been attempted in order to isolate aptamers against intrahepatic carcinomas in mice [53]. In this work, the authors performed intravenous injections of chemically modified RNA pools into tumor bearing mice. Those aptamers that localized to the tumors were extracted and amplified. These efforts resulted in two RNA sequences that target an intracellular, RNA binding protein (P68) overexpressed in the hepatic tumor.

Despite the complex nature of published aptamer selections, the resulting ‘winner’ aptamer sequences are typically few in number and, in most cases, only one sequence is identified and further characterized. The likely reason for the “one selection-one aptamer” phenomenon is that only the most highly represented sequences are sequenced using the traditional chain terminator method, i.e., Sanger sequencing [33], [36], [49]–[52], [56]. Recently, the advent of HTS technologies has streamlined the sequencing process, allowing researchers to obtain a more comprehensive picture of all selected sequences after only a few rounds of selection [8], [18], [45], [46]. Although these published studies were performed against purified, recombinant proteins, the authors observed that the sequences with the highest read number were not necessarily the highest affinity binders [8], [46]. By contrast, high affinity binders had the highest *fold enrichment* during the course of the selection [8], [46]. These findings are in agreement with our data demonstrating that cell-specific internalization of ‘winner’ sequences positively correlated with *fold enrichment*. Interestingly, although we also observed a positive correlation between *fold internalization* and *read number*, this association occurred only after the introduction of a negative selection step. Together, these studies highlight the importance of performing HTS on all rounds of selection in order to assess *fold enrichment* of specific sequences over the course of a selection. In addition, our data identify the negative-selection step as a key criterion to successfully enrich for cell-specific sequences [24].

To date, bioinformatics analyses performed on aptamer selections have relied predominantly on an arbitrary cutoff, typically based on *read number*, to identify aptamers sequences that are subsequently analyzed experimentally [8], [24], [45]. While this strategy has proven effective, candidate sequences with a low read number are have a high probability of being missed or disregarded. In order to identify all possible ‘winner’ sequences, including those that were not highly-represented, we performed pairwise comparisons using *edit distance* and *tree distance* analyses to identify sequences that are related based on sequence or structure. These analyses resulted in the identification of 27 sequences (out of 32) that were among the best cell-specific internalizing aptamers (fold internalization ≥4). Of particular importance, the aptamer with the highest fold internalization (#51) was not among the aptamers with the highest read number. Aptamer #51 was identified only through the *tree distance* analysis, highlighting that RNA structure should also be considered when choosing sequences for experimental validations.

A potential current limitation of the *tree distance* analysis is that it is based on the assumption that each sequence has only a single predicted structure. While RNA molecules can assume multiple breathing dynamics, our data seem to indicate that structural diversity decreases over the course of a selection. However, the inclusion of all possible RNA structures may improve the outcome of current tree distance analyses, though this type of analysis is currently hindered by the existing algorithms for structure prediction. Indeed, we anticipate these multi-structure analyses are likely to become more feasible as predictive RNA structural algorithms evolve and as computational algorithms become more complex.

In conclusion, our studies highlight the utility of combining HTS with bioinformatics analysis for the identification of ‘winner’ sequences from an aptamer selection performed against a complex target. These efforts have yielded (1) predictive tools that are broadly applicable to all aptamer selections for target sequence identification; and (2) several VSMC-specific RNA aptamers that can subsequently be used for targeted delivery. Thus, as the aptamer field enters an exciting new chapter in which HTS and bioinformatics tools become a critical component of aptamer discovery, methodologies such as the one described herein both complement and add to existing approaches for sifting through millions of sequence reads generated from high-throughput sequencing data of aptamer selections.

## Materials and Methods

### Cell Culture

All cell lines were cultured at 37°C under 5% CO_2_. The A7r5 (ATCC, CRL-1444) cell line was cultured in DMEM (Gibco, 11965) supplemented with 10% heat inactivated FBS (Atlanta biologicals, S11550) and the YPEN-1 (ATCC, CRL-2222) cell line was cultured in MEM (Gibco, 11095) supplemented with 5% heat inactivated FBS (Atlanta biologicals, S11550), 1.5 g/L Na bicarbonate (Gibco, 25080), 0.1 mM MEM NEAA (Gibco, 11140), 1.0 mM Na pyruvate (Gibco, 11360) and 0.03 mg/mL heparin (Sigma, H4784). All cell lines were screened for contamination by mycoplasma, which is known to degrade 2′-fluoro pyrimidine modified RNA aptamers [57]. Both cell lines were split 1∶3 or 1∶4 upon reaching confluence by washing with DPBS (Gibco, 14190–144) and detaching cells using 0.25% Trypsin-EDTA (Gibco, 25200) as recommended for each cell line by ATCC. Each cell line was carried for no more than 5–6 passages. For selection rounds and internalization assays, confluent P150 (Nunc, 168381) plates of A7r5 and YPEN-1 were detached as described. Cells were counted by staining an aliquot of cells with 0.4% trypan blue (Gibco, 0618) and using a hemocytometer to count the number of live cells. A7r5 and YPEN-1 cells were plated at 5 million and 8 million cells per P150 respectively for selection rounds and 500,000 and 800,000 cells per well of a 6-well plate respectively for internalization assays. For internalization assay, extra A7r5 and YPEN-1 cells were plated in order to count the number of cells after 24 hrs in culture to normalize internalization assay data to cell number.

### Cell internalization SELEX

#### Generation of the initial (Round 0) RNA Library

The duplex DNA library was generated as follows. The Sel2N20 single stranded DNA (ssDNA) template oligo, 5′-TCGGGCGAGTCGTCTG-N20-CCGCATCGTCCTCCC-3′ (IDT, Coralville, IA) was extended using Choice Taq Polymerase (Denville Scientific Inc., CB4050-2) in the presence of Sel2 5′ primer, 5′-TAATACGACTCACTATAGGGAGGACGATGCGG-3′. The extension reaction was performed in a thermocyler by heating the Sel2N20 ssDNA template oligo and the primer at 95°C for 3 min, annealing at 25°C for 10 min and extending at 72°C for 30 min, followed by a 10 min incubation at 25°C. The duplex DNA library was *in vitro* transcribed for 16 hrs at 37°C using Y639F mutant T7 RNAP [58], [59] to enable incorporation of 2′-OH purines (Roche; GTP, 14611221; ATP, 14919320) and 2′-Fluoro pyrimidines (TriLink BioTechnologies; 2′-Fluoro-2′-dCTP, N-1010-020509; 2′-Fluoro-2′-dUTP, N-1008-013008) in the Round 0 RNA library. During the transcription reaction, the final concentration of rNTPs for targeted selections was 4 mM with a 3∶1 ratio of 2′-F pyrimidines to 2′-OH purines (3 mM 2′-F pyrimidines and 1 mM 2′-OH purines). Duplex DNA was removed from the transcription reaction using DNase I (Roche, 04716728001) and the RNA from the transcription reaction was run on a denaturing gel (10% acrylamide; 7 M urea). The band of RNA was detected by UV shadowing and excised from the gel. The excised gel fragment containing the RNA was incubated with TE buffer to elute the RNA from the gel. Eluted RNA was collected using a 10,000 MWCO centrifugal filter (Amicon Ultra-4, UFC801024) and washed with additional TE buffer. The concentration and purity of the extracted RNA was determined by OD 260/280.

#### Cell-internalization SELEX for VSMC specific internalizing aptamer selection

We applied a modified cell-based SELEX [21] termed cell-internalization SELEX as previously described [24]. A7r5 and YPEN-1 cells were cultured as described under cell culture methods. For each round of selection, 150 nM RNA was folded in Opti-MEM (1500 pmoles in 10 mL) by heating to 65°C for 10 min followed by a 20 min incubation at 37°C. Folded RNA was then supplemented with 100 µg/mL yeast tRNA (Invitrogen). A7r5 and YPEN-1 cells were washed 3× with Opti-MEM (Gibco, 31985) and incubated in Opti-MEM with 100 µg/mL tRNA (Invitrogen, 15401-029) in Opti-MEM for 15 min. For rounds 1 through 3, the RNA library was added to A7r5 cells for 90 min. For rounds 4 through 6, the RNA library was pre-cleared against a single plate of YPEN-1 cells for 15 min. For rounds 7 through 8, the RNA library was pre-cleared against two plates of YPEN-1 for 15 min sequentially. For rounds 4 through 6 and 7 through 8, the unbound RNA (from pre-clear step) was collected, centrifuged (1,500 rpm, 5 min.) to pellet cell debris and added to A7r5 cells for 60 min and 30 min respectively. YPEN-1 pre-clear conditions (time and number of plates) and A7r5 selection conditions (internalization time) are summarized in **Table 1**.

Following incubation of the RNA library, A7r5 cells were washed 3 times with ice cold DPBS to remove unbound RNA. Cell-surface bound RNA was removed by washing cells once with ice cold 0.5 M NaCl DPBS, incubating cells 5 min at 4°C with ice cold 0.5 M NaCl DPBS and washing once with ice cold DPBS. Internalized RNA was recovered by TRIzol (Invitrogen, 15596-026) extraction by following the manufacturer's instructions. The recovered RNA aptamers were reverse transcribed (RT) (Invitrogen, Superscript III, 56575) using the Sel2 3′ primer: 5′-TCGGGCGAGTCGTCTG-3′. The RT protocol is as follows: 55°C for 60 min followed by a 15 min incubation at 72°C. The RT product was PCR-amplified using Choice Taq DNA polymerase (Denville Scientific Inc., CB4050-2) in the presence of the Sel2 5′ and Sel2 3′ primers. The dNTP concentration in the PCR reaction was 2.5 mM (10 mM dNTP mix, Invitrogen, 100004893). The PCR amplification protocol is as follows. 95°C for 2 min, followed by 25 cycles of heating to 95°C for 30 sec, 55°C for 30 sec and 72°C 30 sec. A final extension step was performed for 5 min at 72°C. The DNA duplex library was *in vitro* transcribed as described above.

### DNA melt assay

We used a modified DiStRO method [42] DNA melt assay to determine library complexity. SYBR green (BioRad, 170-8882) was added at a 1∶1 volume to 0.5 µM DNA duplex library of each selection round in triplicate. These samples were run on a real-time PCR machine (Eppendorf, Mastercycler epgradient S with realplex^2^) using a DNA melt assay protocol (95°C for 15 min; 95°C for 15 sec; 95°C–25°C ramp for 20 min and 25°C for 15 sec; 4°C hold). The raw DNA melt assay data (performed in triplicate) was averaged and plotted as fluorescence intensity (SYBR) *vs*. temperature (C°). Higher library complexity was indicated by a relative shift of the DNA melt curve towards lower temperatures, whereas, lower library complexity was a shift to higher temperatures.

### Internalization assay by quantitative reverse transcription-PCR (RT-qPCR)

We applied similar RT-qPCR methods to detect internalized RNA as described previously [24], [60]. A7r5 and YPEN-1 cells were incubated with 150 nM RNA aptamer library (rounds 0 through 8) or 150 nM RNA aptamer for 30 min. at 37°C with 5% CO_2_. Unbound and cell-surface bound RNA was removed by washing with ice-cold 0.5 M NaCl DPBS. Internalized RNA was recovered by TRIzol containing 0.5 pmoles/mL M12–23 aptamer [61] as a reference control. The amount of recovered RNA was determined by performing a two-step RT-qPCR protocol. In Step one, recovered RNA was reverse transcribed using MMuLV, NEB, M0253L. The primers for the RT step were either the Sel2 3′ primer or M12–23 aptamer reference control primer (5′- GGGGGGATCCAGTACTATCGACCTCTGGGTTATG -3′). The RT protocol is as follows. The recovered RNA and the primers were heated at 65°C for 5 min, annealed at 22°C for 5 min and extended at 42°C for 30 min followed by and extension at 48°C for 30 min. In Step two, the product from the RT reaction was PCR amplified with iQ SYBR Green Supermix (BioRad, 170-8882) using a Eppendorf Mastercycler epgradient S with realplex^2^. The qPCR protocol is as follows. The RT product was heated at 95°C for 2 min, followed by 50 cycles of heating at 95°C for 30 sec, annealing at 55°C for 30 sec, and extending at 72°C for 30 sec. A melt curve was performed by heating at 60°C–95°C for 20 min. Reactions were all done in 50 µL volume in triplicate with either the Sel2 5′ and 3′ primers or M12–23 aptamer reference control 5′ (5′- GGGGGAATTCTAATACGACTCACTATAGGGAGAGAGGAAGAGGGATGGG -3′) and 3′ primers. Data were normalized to the M12–23 reference control (**Figure S2**) and to cell number as determined by counting cells cultured in conjunction with each experiment.

### Illumina high-throughput sequencing sample preparation

#### Illumina high-throughput sequencing sample preparation

The RNA pools for selection rounds 0 (in duplicate), 1, 3, 5, 6, 7 and 8 were reverse transcribed using Superscript III (Invitrogen, 56575) in the presence of the Sel2 3′ primer. The RT protocol is as follows. The round RNA was heated at 55°C for 60 min and extended at 72°C for 15 min. The product from the RT reaction was PCR amplified using Choice Taq DNA Polymerase (Denville Scientific Inc., CB4050-2) in the presence of Illumina primers (5′-AATGATACGGCGACCACCGAGATCTACACTCTTTCCCTACACGACGCTCTTCCGATCT-8 nt Barcode-GGGAGGACGATGCGG-3′; 5′-CAAGCAGAAGACGGCATACGAGCTCTTCC GATCTTCGGGCGAGTCGTCTG-3′). The PCR Protocol is as follows. The RT product was heated at 95°C for 2 min, followed by 10 cycles of heating at 95°C for 30 sec, annealing at 55°C for 30 sec and extending at 72°C for 30 sec and a final extension step at 72°C for 5 min. The PCR product was run on a 2.5% agarose gel and the appropriate band (∼151 bp) was excised, gel purified and quantitated using a UV spectrophotometer (OD 260). Samples were combined at equal molar amounts and submitted for Illumina sequencing (Iowa State University DNA Facility, Ames, IA.; Illumina Genome Analyzer II).

#### Illumina high-throughput sequencing data pre-processing

The Illumina base calls were pre-processed and filtered to identify the variable region sequence as previously described [24], [48]. These data (total sequences) included all variable region sequences, including replicates, for a given round of selection. The total sequences were collapsed to individual sequences with total associated reads for each individual sequence. These data (unique sequences) include the different variable region sequences for a given round and the *read number* (cluster size) associated with each sequence. The unique sequences and total sequences were then converted from DNA code to RNA code and the 5′/3′ constant regions were added to give the full length RNA aptamer sequence. Table S1 summarizes the Illumina data obtained and the data filtered during pre-processing (**Materials S1 and Table S1**).

### Bioinformatics analyses

#### Sequence complexity and sequence enrichment

Sequence complexity was determined using the equation; sequence complexity = unique sequences/total sequences. Sequence enrichment was calculated by taking the complement of % sequence complexity using the equation; sequence enrichment = 1-(unique sequences/total sequences). Unique sequences and total sequences refer to the data obtained during Illumina high-throughput sequencing data pre-processing.

#### Minimum free energy, ensemble free energy, ensemble probability and ensemble diversity

The set of unique sequences found within each round were analyzed with RNAfold [43], [62]–[64] (-T 30, -noLP, -noGU, -d2) from Vienna Package v2.0.0 [43], [44]. A program (process_seqs_rnaFold) was created to allow for batch processing of sequences with RNAfold and included RNAfold data output in a comma delimited format. The average and SEM for minimum free energy (kcal/mol), ensemble free energy (kcal/mol), ensemble probability (Probability, %) and ensemble diversity (Diversity, #) were calculated. For the set of total sequences the averages and SEM were recalculated using the *read number* for each unique sequence.

#### Variable region length, frequency of sequences between rounds and frequency of cluster sizes

A non-redundant database was created of all unique sequences found within the rounds of selection (rounds 0, 1, 3, 5, 6, 7, 8). For each sequence, this database tracked the variable region nucleotide (nt) length (minimum = 1 nt, maximum = 56 nt), the number of rounds the sequence was identified in (minimum = 1 round; maximum = 7 rounds) and the *read number* (cluster size) of the sequence (minimum = 1 read, maximum = 956,532 reads). Using this database; the average, SEM, mode, maximum, minimum and frequency of the variable region nucleotide length was determined for each round; the frequency of the number of rounds sequences were found was determined for round 0 and rounds 1 through 8; the frequency of cluster sizes was determined for round 0 and rounds 1 through 8.

#### Sequence families and structure families

A program (process.seqs) was created that used RNAfold and RNAdistance from the Vienna Package (v 2.0.0) [43], [44] to first predict the most likely structure and to second determine the edit/tree distance of all sequences/structures to each other. The program process.seqs filtered these data using a predefined limit on either edit distance (**Figure S3**; data output for edit distance = 1) or tree distance (**Figure S4**; data output for tree distance = 3). Process.seqs was run using increasing values of edit/tree distance to determine the maximum edit/tree distance where all sequences/structures connected. Clusters of sequences/structures interconnected at each edit/tree distance were determined and separated using the program Cytoscape (v 2.8.1). Using these data, a dendrogram of sequence/structure relatedness by edit/tree distance was created and the resulting dendrogram was evaluated using Cytoscape. The edit/tree distance dendrogram indicates the edit/tree distance that the unique sequences interconnect. Sequence/structure families were evaluated at each edit/tree distance using the multiple sequence alignment program ClustalX (v 2.1) or multiple RNA structure alignment program LocARNA (webserver).

#### Calculations for rising, fold enrichment and rate enrichment

The previously described non-redundant database was used to normalize the *read number* of each sequence from a given round to the total reads from all rounds. To avoid dividing by 0 and simplify calculations, a *read number* of 1 was assumed with sequences with a *read number* of 0. The *rising*, fold enrichment and rate enrichment was determined for all unique sequence between rounds 1 through 3, 1 through 8, 3 through 8 and 6 through 8. ‘*Rising*’ was determined by calculating the correlation coefficient (−1.0 through 1.0) for each sequence using the *read number* and round number. Fold enrichment was determined by dividing the number of reads in a given rounds with the *read number* in a previous round. Rate enrichment was calculated by dividing the change in number of reads by the change in round number.

### Plotting and statistics

Data were plotted using either Microsoft Excel 2010 or GraphPad Prism 5. Average, SEM, mode and correlation were determined using Microsoft Excel 2010. Curve fitting was done using GraphPad Prism 5. Student's t-test was done using Microsoft Excel 2010 with significance set at a *p* value of <0.05. Correlation coefficients were determined using GraphPad Prism 5 by first determining normality and then the Spearman's correlation coefficient. Significance of the correlation coefficient was determined at a two-tailed *p*-value of <0.05.

## Supporting Information

Figure S1 **Amount of RNA aptamer library (Round 0) internalized into VSMCs over time.** Round 0 RNA was incubated with VSMCs (A7r5) for 15, 30, 60, 90 or 120 minutes. Unbound RNA or RNA bound to the surface of cells was removed with a stringent salt wash. Internalized RNA was extracted by TRIzol extraction and measured using RT-qPCR.
(TIF)

Figure S2 **Recovery of RNA processing control.** The M12–23 reference control RNA was added to TRIzol prior to cell lysis as a control for processing of internalized RNA. The reference control M12–23 RNA was measured by RT-qPCR. Internalized RNA data was normalized to each paired processing M12–23 reference control RNA.
(TIF)

Figure S3 **Edit distance = 1 output from process.seqs program.** Unique sequences (black nodes) interconnect by edges (lines) at edit distance 1 (blue lines). This analysis resulted in several sequence clusters as well as sequences that did not fall within a sequence cluster (individual nodes not interconnected by blue lines). Different unique sequences within a sequence cluster may have edit distances greater than 1, but are linked by intervening unique sequences separated by no more than 1 edit distance (blue lines). Cytoscape was used to assign the unique sequences of each separate cluster a number. This data was used to generate the dendrogram in Figure 4A. This edit distance analysis was repeated for edit distances 2–9, where at edit distance = 9 only one cluster of interconnect unique sequences was generated.
(TIF)

Figure S4 **Unique sequences interconnected by a tree distance = 3.** Structures of unique sequences (black nodes) interconnect by edges (lines) of tree distances 0–3 (0 = blue lines, 1 = cyan lines, 2 = green lines, 3 = red lines). This analysis resulted in several structure clusters as well as structures of unique sequences that did not fall within a structure cluster (individual nodes not interconnected by any colored lines). Different unique sequences within a structure cluster may have tree distances greater than 3, but are linked by intervening structures separated by no more than 3 tree distances (blue, cyan, green and red lines). Cytoscape was used to assign the unique sequences of each separate structure cluster a number. This data was used to generate the dendrogram in Figure 4B. This tree distance analysis was repeated for tree distances 4–22, where at tree distance = 22 only one structure cluster of interconnect unique sequences was generated.
(TIF)

Figure S5 **Binding and internalization of Fam-GTP labeled aptamers.** RNA aptamers #51 and #420 were labeled during *in vitro* transcription with a Fam-GTP. Binding of aptamers #51 and #420 was determined by fluorescence after several PBS washes without high salt. Internalization of aptamer #51 was determined at 150 nM and 300 nM by fluorescence following several PBS washes that included a high salt wash.
(TIF)

Table S1 **Summary of high-throughput sequence data.**
(DOC)

Materials S1 **Supplementary Information.** Methods are presented for the binding and internalization assays performed using fluorescent-labeled RNA aptamers (FAM-G-aptamers). Corresponding reference citations for this assay are also presented.
(DOCX)

Database S1 **Comma separated values (.CSV) database includes all unique selected sequences (2,312).** Column label identifiers after importing into excel: **A** (*#*) = identification number. **B** (*Representatives*) = selected RNA aptamer representatives (X). **C** (*HTS read variable region*) = variable region identified by HTS. **D** (*Length*) = nucleotide length of the variable region. **E** (*1*) = number of reads in round 1. **F** (*3*) = number of reads in round 3. **G** (*5*) = number of reads in round 5. **H** (*6*) = number of reads in round 6. **I** (*7*) = number of reads in round 7. **J** (*8*) = number of reads in round 8. **K** (*Total*) = total number of reads in rounds 1–8. **L** (*Rounds*) = number of rounds. **M** (*RNA*) = RNA aptamer sequence (variable region with 5′ and 3′ constant regions). **N** (*Structure*) = predicted secondary structure of the RNA aptamer sequence. **O** (*Sequence*) = sequence family. **P** (*Structure*) = structure family.
(CSV)
