# Supplementary figures and images for: Rapid Identification of Cell-Specific, Internalizing RNA Aptamers with Bioinformatics Analyses of a Cell-Based Aptamer Selection
Source: PLoS One. 2012 Sep 4;7(9):e43836. doi: 10.1371/journal.pone.0043836 (PMC3433472; doi:10.1371/journal.pone.0043836)

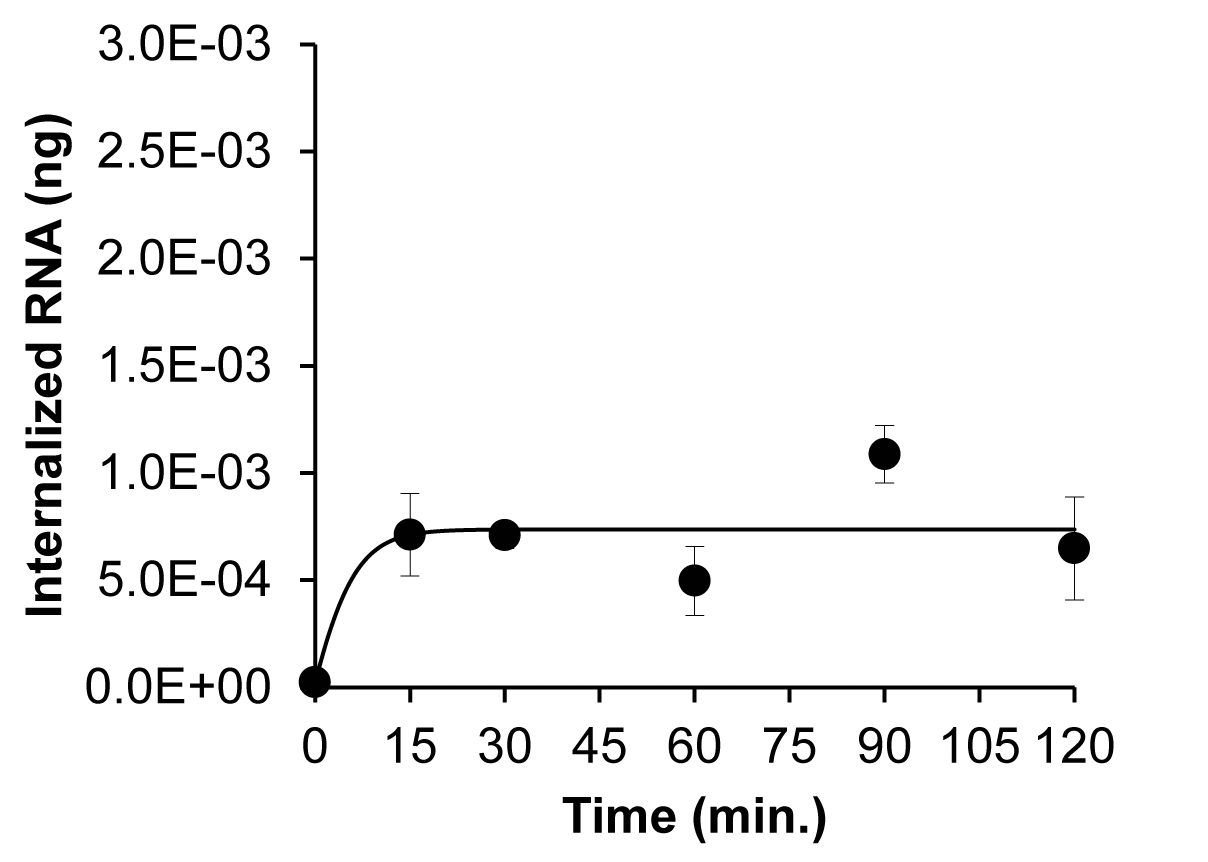

Supplement: Figure S1 — Amount of RNA aptamer library (Round 0) internalized into VSMCs over time. Round 0 RNA was incubated with VSMCs (A7r5) for 15, 30, 60, 90 or 120 minutes. Unbound RNA or RNA bound to the surface of cells was removed with a stringent salt wash. Internalized RNA was extracted by TRIzol extraction and measured using RT-qPCR. (TIF) [file pone.0043836.s001.tif]

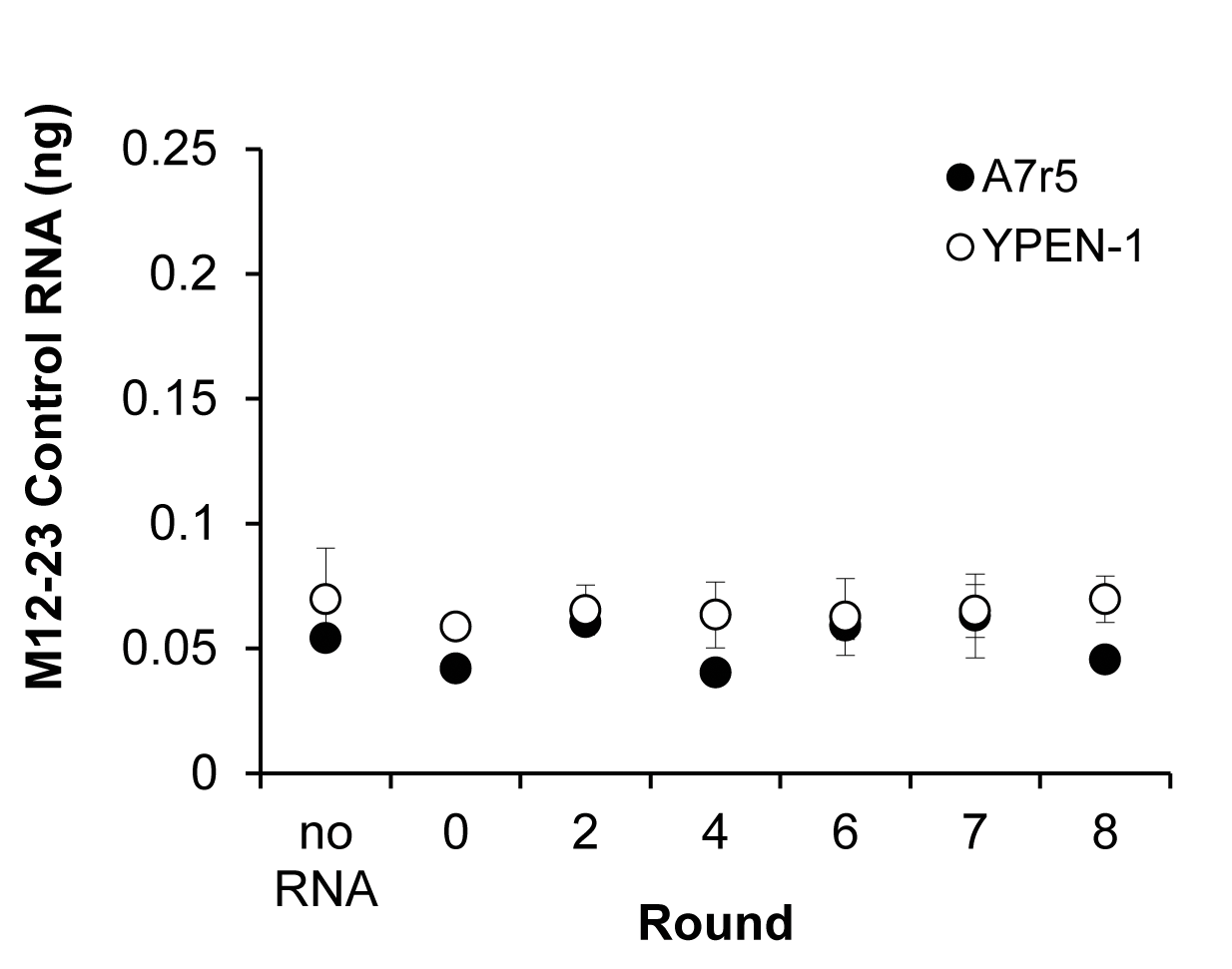

Supplement: Figure S2 — Recovery of RNA processing control. The M12–23 reference control RNA was added to TRIzol prior to cell lysis as a control for processing of internalized RNA. The reference control M12–23 RNA was measured by RT-qPCR. Internalized RNA data was normalized to each paired processing M12–23 reference control RNA. (TIF) [file pone.0043836.s002.tif]

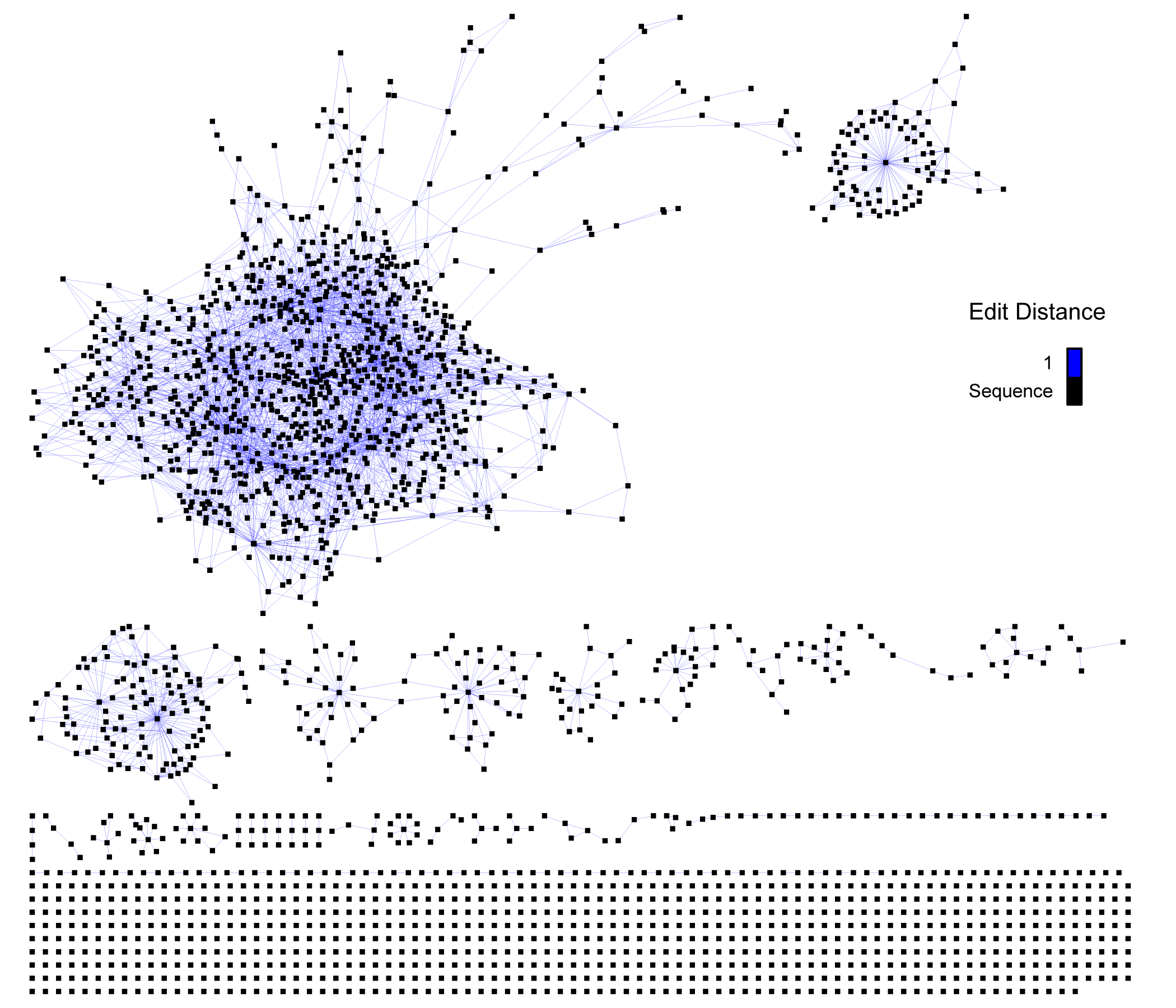

Supplement: Figure S3 — Edit distance = 1 output from process.seqs program. Unique sequences (black nodes) interconnect by edges (lines) at edit distance 1 (blue lines). This analysis resulted in several sequence clusters as well as sequences that did not fall within a sequence cluster (individual nodes not interconnected by blue lines). Different unique sequences within a sequence cluster may have edit distances greater than 1, but are linked by intervening unique sequences separated by no more than 1 edit distance (blue lines). Cytoscape was used to assign the unique sequences of each separate cluster a number. This data was used to generate the dendrogram in Figure 4A. This edit distance analysis was repeated for edit distances 2–9, where at edit distance = 9 only one cluster of interconnect unique sequences was generated. (TIF) [file pone.0043836.s003.tif]

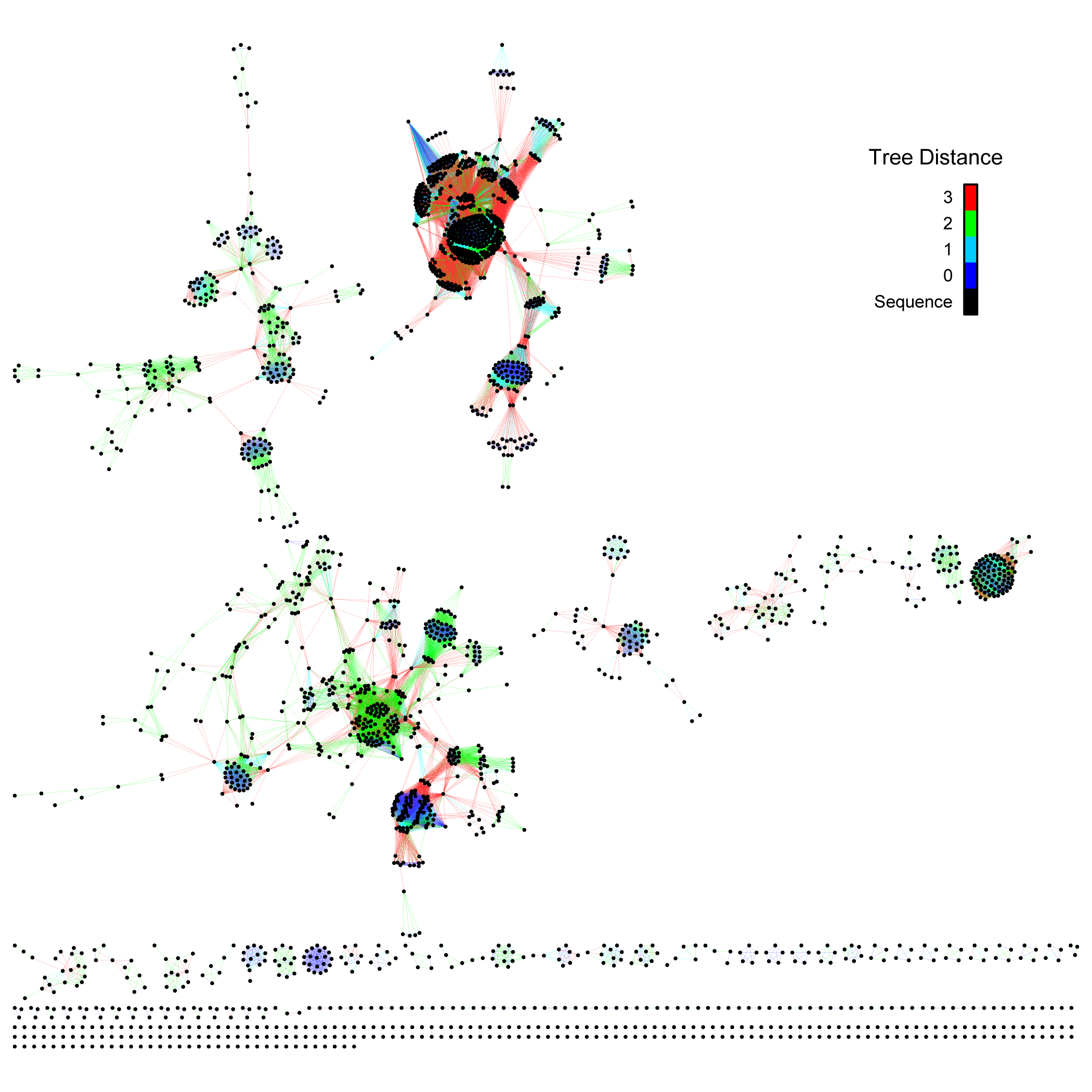

Supplement: Figure S4 — Unique sequences interconnected by a tree distance = 3. Structures of unique sequences (black nodes) interconnect by edges (lines) of tree distances 0–3 (0 = blue lines, 1 = cyan lines, 2 = green lines, 3 = red lines). This analysis resulted in several structure clusters as well as structures of unique sequences that did not fall within a structure cluster (individual nodes not interconnected by any colored lines). Different unique sequences within a structure cluster may have tree distances greater than 3, but are linked by intervening structures separated by no more than 3 tree distances (blue, cyan, green and red lines). Cytoscape was used to assign the unique sequences of each separate structure cluster a number. This data was used to generate the dendrogram in Figure 4B. This tree distance analysis was repeated for tree distances 4–22, where at tree distance = 22 only one structure cluster of interconnect unique sequences was generated. (TIF) [file pone.0043836.s004.tif]

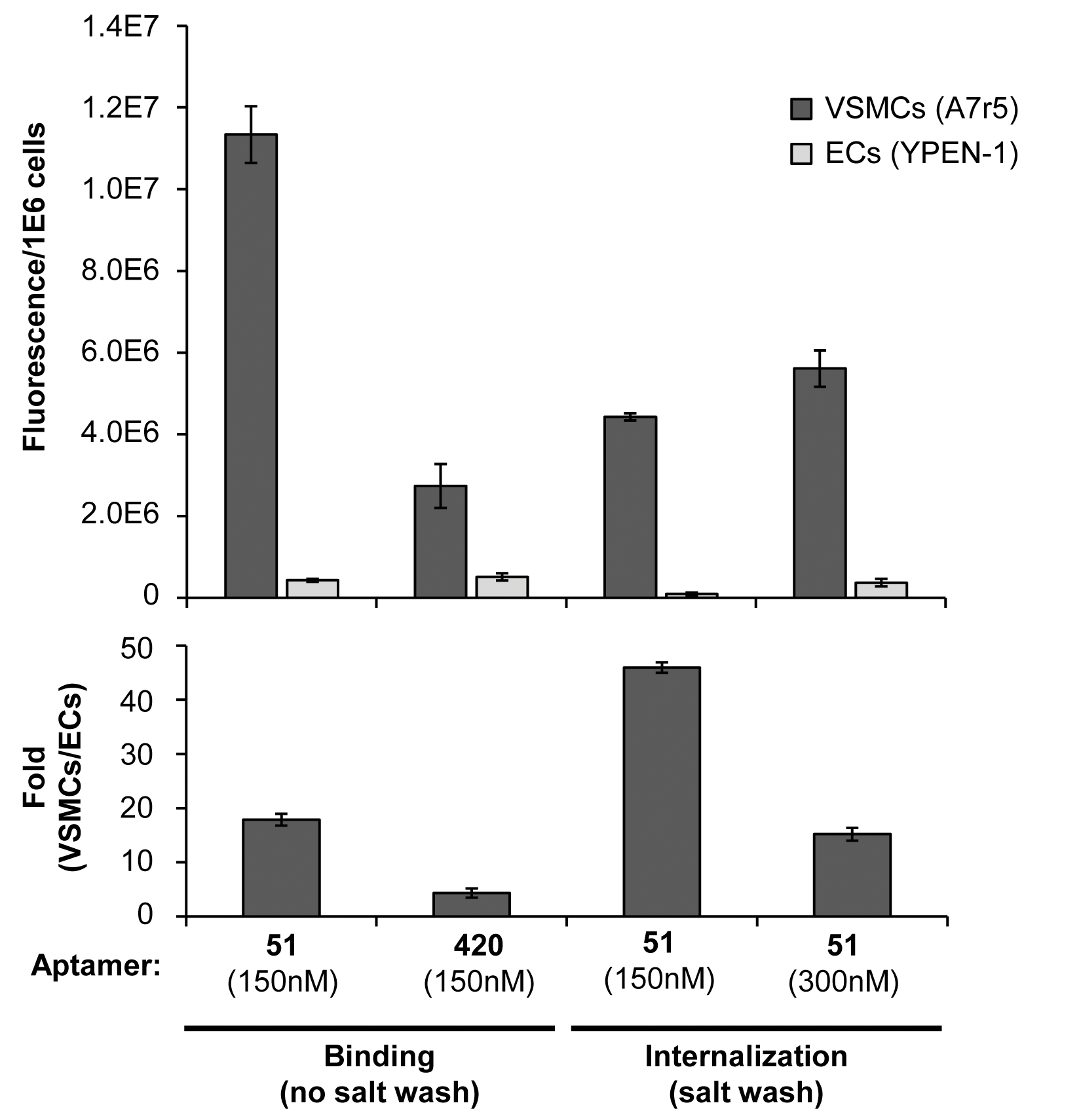

Supplement: Figure S5 — Binding and internalization of Fam-GTP labeled aptamers. RNA aptamers #51 and #420 were labeled during in vitro transcription with a Fam-GTP. Binding of aptamers #51 and #420 was determined by fluorescence after several PBS washes without high salt. Internalization of aptamer #51 was determined at 150 nM and 300 nM by fluorescence following several PBS washes that included a high salt wash. (TIF) [file pone.0043836.s005.tif]
