# Supplementary material for: Rapid Identification of Cell-Specific, Internalizing RNA Aptamers with Bioinformatics Analyses of a Cell-Based Aptamer Selection
Source: PLoS One. 2012 Sep 4;7(9):e43836. doi: 10.1371/journal.pone.0043836 (PMC3433472; doi:10.1371/journal.pone.0043836)
Supplement: Table S1 — Summary of high-throughput sequence data. (DOC) [file pone.0043836.s006.doc]

**Table S1: High-throughput sequencing data summary**

| **Round** | **Unfiltered**  **(#)** | **Removed**  **(#)** | **Filtered**  **(%)** | **Total Reads**  **(#)** | **Unique Reads**  **(#)** | **Sequence**  **Enrichment (%)** |
| --- | --- | --- | --- | --- | --- | --- |
| **0** | 285,103 | 5,775 | 2.03 | 279,328 | 278,929 | 0.14 |
| **0** | 528,852 | 11,405 | 2.16 | 517,447 | 516,475 | 0.19 |
| **1** | 366,528 | 8,856 | 2.42 | 357,672 | 353,246 | 1.24 |
| **3** | 286,540 | 7,016 | 2.45 | 279,524 | 224,842 | 19.56 |
| **5** | 319,642 | 6,806 | 2.13 | 312,836 | 33,259 | 89.37 |
| **6** | 321,715 | 6,940 | 2.16 | 314,775 | 8,264 | 97.37 |
| **7** | 260,986 | 6,330 | 2.43 | 254,656 | 6,836 | 97.32 |
| **8** | 235,673 | 5,141 | 2.18 | 230,532 | 4,113 | 98.22 |
| **Total** | 2,605,039 | 58,269 | 2.23 | 2,546,770 | 1,425,964 |  |
