# Supplementary material for: Rapid Identification of Cell-Specific, Internalizing RNA Aptamers with Bioinformatics Analyses of a Cell-Based Aptamer Selection
Source: PLoS One. 2012 Sep 4;7(9):e43836. doi: 10.1371/journal.pone.0043836 (PMC3433472; doi:10.1371/journal.pone.0043836)
Supplement: Materials S1 — Supplementary Information. Methods are presented for the binding and internalization assays performed using fluorescent-labeled RNA aptamers (FAM-G-aptamers). Corresponding reference citations for this assay are also presented. (DOCX) [file pone.0043836.s007.docx]

**MATERIALS S1**

**METHODS**

**Binding and internalization assay by fluorescence of Fam-G labeled RNA aptamers**

We used a modified version of the quantitative uptake method previously described [[1](#_ENREF_1)]. Fam-G labeled RNA was *in vitro* transcribed with the addition of 3mM 6-FaM GTP (TriLink BioTechnologies) and purified using the same methods as previously described [[2](#_ENREF_2),[3](#_ENREF_3)]. Cells (A7r5 and YPEN-1) were prepared and blocked with tRNA as described for under the internalization assay by RT-qPCR methods. A “no RNA” control was included for each cell type to determine background fluorescence of the cell lysate. The Fam-G labeled RNA aptamers were folded in OPTI-MEM at 150nM using the following modified RNA folding protocol; 98°C for 10 min.; 65°C for 15 min.; 37°C for 20 min. The tRNA block was discarded and Fam-G labeled aptamers were added to cells for 30 minutes. Following RNA aptamer incubation, all samples, including the “no RNA” control, were washed 4x using one of two protocols to determine either binding or internalization. Washes for *binding assay* (no salt wash): 2x ice cold DPBS, 1x ice cold DPBS with 5 min. 4°C incubation, 1x ice cold DPBS. Washes for *internalization assay* (salt wash): 1x ice cold DPBS, 1x ice cold 0.5M NaCl DPBS, 1x ice cold 0.5M NaCl DPBS with 5 min. 4°C incubation, 1x ice cold DPBS. Following washes all samples had 600uL 0.1M NaOH added to lyse cells. During lysis, the cell number control was collected and counted for each cell type as described previously. Cell lysate was collected using a cell scraper into 1.5mL microcentrofuge tubes. Genomic DNA was sheared by sonication, level 2 for 10 seconds (Fisher Scientific Sonic Dimembrator Model 100). Insoluble protein was pelleted by centrifugation for 10 minutes at 10,000g at room temperature. Lysate was aliquoted (100uL) into a black 96-well plate (Nunc #237105) and fluorescence (LjL BioSciences Analyst HT 96.384; Fluorescein Protocol: 1mm depth, 485-20 excitation, 530-25 emission) of each sample was determined. Fluorescence data was analyzed by subtracting background fluorescence measured from the “no RNA” control, normalizing to cell number, calculating the average and calculating the SEM.

**REFERENCES**

1. Lundberg P, El-Andaloussi S, Sutlu T, Johansson H, Langel U (2007) Delivery of short interfering RNA using endosomolytic cell-penetrating peptides. FASEB J 21: 2664-2671.

2. Thiel KW, Hernandez LI, Dassie JP, Thiel WH, Liu X, et al. (2012) Delivery of chemo-sensitizing siRNAs to HER2+-breast cancer cells using RNA aptamers. Nucl Acids Res.

3. McNamara JO, Andrechek ER, Wang Y, Viles KD, Rempel RE, et al. (2006) Cell type–specific delivery of siRNAs with aptamer-siRNA chimeras. Nature Biotechnology 24: 1005-1015.
